# Generative Transfer Learning for Intelligent Fault Diagnosis of the Wind Turbine Gearbox

**DOI:** 10.3390/s20051361

**Published:** 2020-03-02

**Authors:** Jianwen Guo, Jiapeng Wu, Shaohui Zhang, Jianyu Long, Weidong Chen, Diego Cabrera, Chuan Li

**Affiliations:** 1 School of Mechanical Engineering, Dongguan University of Technology, Dongguan 523808, China; guojw@dgut.edu.cn (J.G.); jiapengw95@163.com (J.W.); longjy@dgut.edu.cn (J.L.); chuanli@dgut.edu.cn (C.L.); 2 School of Electromechanical Engineering, Guangdong University of Technology, Guangzhou 510006, China; 3 Institute of High Energy Physics, CAS, Dongguan 523803, China; chenwd@ihep.ac.cn; 4 GIDTEC, Universidad Politécnica Salesiana, Cuenca 010102, Ecuador; dcabrera@ups.edu.ec

**Keywords:** domain adaptation, adversarial training, GAN, wind turbine gearbox

## Abstract

Intelligent fault diagnosis algorithms based on machine learning and deep learning techniques have been widely used in industrial applications and have obtained much attention as well as achievements. In real industrial applications, working loads of machines are always changing. Hence, directly applying the traditional algorithms will cause significant degradation of performance with changing conditions. In this paper, a novel domain adaptation method, named generative transfer learning (GTL), is proposed to tackle this problem. First, raw datasets were transformed to time–frequency domain based on short-time Fourier transformation. A domain discriminator was then built to distinguish whether the data came from the source or the target domain. A target domain classification model was finally acquired by the feature extractor and the classifier. Experiments were carried out for the fault diagnosis of a wind turbine gearbox. The t-distributed stochastic neighbor embedding technique was used to visualize the output features for checking the effectiveness of the proposed algorithm in feature extraction. The results showed that the proposed GTL could improve classification rates under various working loads. Compared with other domain adaptation algorithms, the proposed method exhibited not only higher accuracy but faster convergence speed as well.

## 1. Introduction

Nowadays, maximizing the use of clean energy is crucial due to the significant increase in energy consumption. As one of the clean energy sources, wind energy has received growing attention. Wind turbines, which turn wind energy into electrical energy, are frequently used in the industrial field. However, a possible consequence of the increase in wind turbine use is that the economic losses will be more significant should they break down. Therefore, to ensure windmill generators working in a reliable and safe environment, fault diagnosis for wind turbines is inevitable.

Data-driven fault diagnosis methods, which can effectively make use of statistical features of massive amounts of collected data and give a reliable result based on these features, are the most potent machinery fault detection techniques. Typical intelligent fault diagnosis has two main steps: feature extraction and faulty mode recognition. In order to achieve high recognition accuracy, a lot of effort has been made on feature extraction. Zhang et al. [1] used permutation entropy values of a vibration signal decomposed into a set of intrinsic mode functions by ensemble empirical mode decomposition to extract fault features and then fed the fault features into an optimized support vector machine (SVM) to get good results. Wang et al. [2] proposed a new fault diagnosis model for H-bridge multilevel inverter based on fast Fourier transform (FFT), relative principle component analysis, and SVM. Li et al. [3] proposed a new feature extraction and evaluation method to obtain the statistical features of vibration signals of rotating machinery and approximate normal distributions. After that they used a classifier to distinguish the fault pattern. A step-by-step compound fault diagnosis method was reported in [4].

Although all the studies above using intelligent fault diagnosis have shown fair performance, there are two shortcomings: (1) the feature extraction step depends heavily on human experience in signal preprocessing, and (2) they perform poorly under varying working conditions.

To overcome these problems, end-to-end deep learning structures, such as convolutional neural network (CNN) [5,6], echo state network [7,8,9], extreme learning machine [10] and sparse autoencoder [11,12,13], have been proposed and have drawn much attention in machinery fault diagnosis. Moreover, the optimization algorithms [14,15,16] in machine learning also keep pace with the times. With the forward and back propagation procedure, these deep structures gain powerful ability for machinery fault detection.

However, there are still difficulties for real-world applications. The critical point of deep learning methods is collected data with labels. However, in real-world applications, labelling data is both time- and money-consuming [17]. Thus, transfer learning, a new branch of deep learning, has come up to solve this problem. It tries to build a model using little target domain data with or without labels [18] based on knowledge transfer. Recently, transfer learning, mainly the domain adaptation branch, has been applied to various fields and achieved excellent results. It has also gained extensive attention in the field of fault monitoring and diagnosis [19,20,21,22]. In powerful deep structure applications, preprocess is not required. However, for practical interpretation of engineering machine learning, some preprocessing treatments are needed during detection model building, such as time–frequency transformation [23], autocorrelation power spectrum [24], FFT [25], etc.

Currently, generative adversarial network (GAN) is one of the most popular networks, which was proposed by Goodfellow et al. [26]. It contains generator *G* and discriminator *D*, where *G* is for capturing data distribution, while *D* is for estimating the probability of whether a sample comes from the real world or from *G*. Ganin et al. [27] employed the adversarial mechanism in transfer learning by adding a gradient reverse layer. Diego et al. [28] applied GAN to tackle the problem of data imbalance in reciprocating machinery fault diagnosis. Inspired by GAN, we designed a domain adaptation model based on GAN, named the generative transfer learning (GTL) method. The main aim of this work was to (1) design an auto feature extractor without prior experience that can reach high performance and normalize the feature extraction procedure and (2) introduce GAN into the domain adaptation method and take the target model as a generator to learn the distribution of target domain data, which will reduce large amounts of training costs.

The rest of this paper is organized as follows. In Section 2, theoretical works of the proposed approach are provided. The intelligent fault diagnosis experimental settings based on the GTL model are outlined in Section 3. The results of the experiments are analyzed in Section 4. Finally, the conclusion and future expectations based on our work are addressed in Section 5.

## 2. Methodology

In this section, the basics of the data preprocessing method are introduced in the first subsection. Aimed at describing the data flow in the model, a convolutional neural network with batch normalization (BN) is reported in the second subsection. The adversarial discriminative domain adaptation method is introduced in the third subsection. Finally, a flowchart giving an overview of the proposed algorithm is introduced in the last subsection.

### 2.1. Overview of the Proposed Algorithm

**Figure 1:**
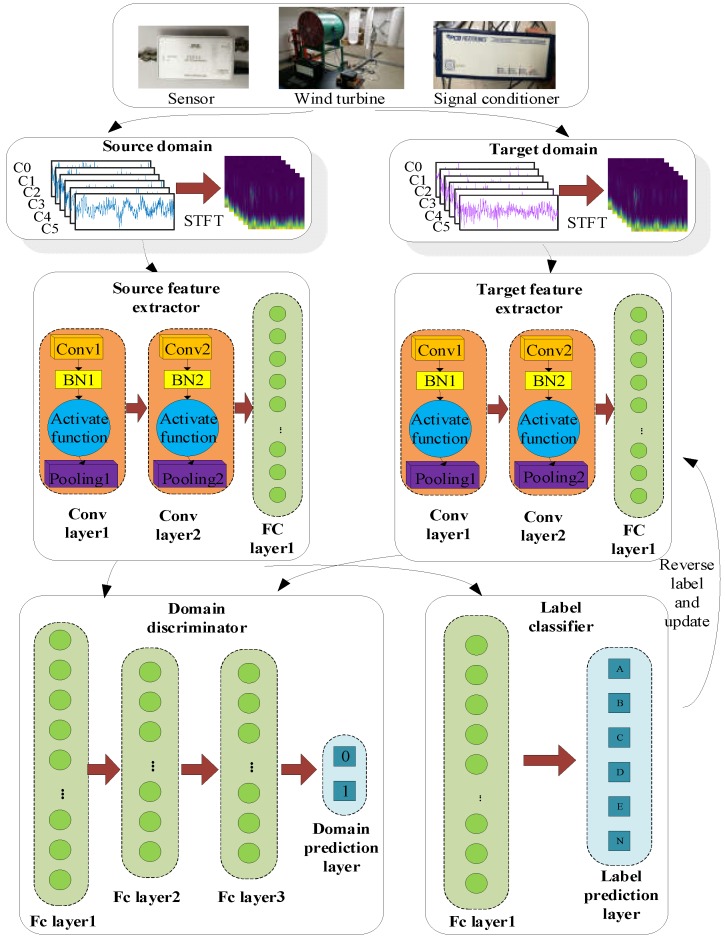
Schematic of the proposed generative transfer learning (GTL) method.

The optimization of the whole algorithm can be divided into three parts: a source feature extractor and a source classifier model establishment; adversarial discriminative domain adaptation; and a target feature extractor combined with the source classifier. The algorithm for the proposed GTL method is illustrated in Figure 1 and also summarized below.

**Step 1**. Collect raw signal from the accelerometer installed on the rotating machinery.

**Step 2**. Perform short-time Fourier transformation (STFT) and change the raw data from the time domain into the time–frequency domain.

**Step 3**. Build a source domain classification model containing a source feature extractor and a source label classifier.

**Step 4**. Build a domain discriminator to distinguish whether the data came from the source or the target domain, with source data labeled as 0 and target data labeled as 1.

**Step 5**. Build a target feature extractor that is the same architecture as the source feature extractor and upgrade it after domain discriminator using reverse labels.

**Step 6**. After the whole adaptation procedure, the target domain classification model is constructed by combining the target feature extractor and the source label classifier.

### 2.2. Data Preprocessing

As one of the most common technologies in digital signal processing, STFT has been widely used in industrial applications. STFT was proposed to make up for the limitations in analyzing the frequency domain features of Fourier transformation. By combining the time domain analysis and frequency domain analysis in time-series signals, the results of STFT can represent both time and frequency domains. The main procedure of STFT involves first using a window function that multiplies with the time signal and performs Fourier transformation in the window and then obtaining the instantaneous spectrum of the signal. After that, the interception window is moved along the time axis, and the spectrum of the whole-time domain is obtained.

The samples fed into the model can be represented as

(1) $$x^{i}=\sum_{n=-\infty }^{\infty }sig(n^{i})w(n-m)e^{-jwn}$$

where *x^i^*∈R^n*n^ denotes the *i*-th generated sample, and *n^i^* is the *i*-th raw signal.

### 2.3. Convolutional Neural Network with Batch Normalization

Convolutional neural network, one of the most powerful deep learning structures, contains two parts: filter and classifier. The filter consists of four kinds of layers: convolutional layer, batch normalization layer, activation function, and pooling layer. Furthermore, the classifier is a multilayer perceptron composed of several fully connected layers.

The traditional architecture of a CNN filter contains three parts: convolutional layer, activation function, and pooling layer. The convolutional layer extracts features by convolving the local input regions with filter kernels. Activation function is key for the model to acquire the ability of nonlinear expression. The pooling layer can perform the downsampling procedure to maintain vital features to reduce the parameters of the network. In this study, the batch normalization layer was employed for reducing internal covariate shift while accelerating the training process of a deep network [29].

The transformation of the BN layer can be represented by

(2) $${\hat{y}}^{l(i,j)}=\frac{y^{l(i,j)}-\mu_{B}}{\sqrt{\sigma_{B}^{2}+\varepsilon }}$$

(3) $$z^{l(i,j)}=\gamma^{l(i)}{\hat{y}}^{l(i,j)}+\beta^{l(i)}$$

where *y^l^*^(*i,j*)^ is the output of the convolutional layer, *μ_B_* is the mean of *y^l^*^(*i,j*)^, and *σ_B_^2^* is the variance of *y^l^*^(*i,j*)^; *ε* is a small constant to avoid the denominator equaling 0. Furthermore, *z^l^*^(*i,j*)^ is the output of one neuron response, *γ^l^*^(*i*)^ and *β^l^*^(*i*)^ are scale parameter and shift parameter, respectively.

For the empirical application, the BN layer is always added between the convolutional layers and the activation function in the filter part. In the classifier part, the BN layer is added right after the fully connected layer and before the activation unit. It is important to note that the BN layer should not be added in the classifier part when it has less than three layers, in case the setting brings some bad results.

### 2.4. Adversarial Discriminative Domain Adaptation Based on GAN

GAN is one of the most popular architectures in deep learning. It introduces the concept of confrontation between two networks, i.e., generator network (*G*) and discriminator network (*D*), to make the data generated by the generator network infinitely approximate the original data in distribution.

Given the real data distribution *P_r_*(*x*), the random noise *z*, and the generated data distribution *P_G_*(*x;θ*) = *G*(*P_z_*), one may use maximum likelihood estimation to find *θ*. This can force *P_G_*(*x; θ*) to approximate *P_r_*(*x*). The equation can be written as

(4) $$L=\prod_{i=1}^{m}P_{G}(x^{i};\theta )$$

where *m* is the number of samples. *θ* can be calculated from

(5) $$\begin{array}{cc} \theta^{*} & =\arg\underset{\theta }{\max}\sum_{i=1}^{m}\log P_{G}(x^{i};\theta )\\ & \approx \arg\underset{\theta }{\max}E_{x\sim P_{r}}[\log P_{G}(x;\theta )]\\ & =\arg\underset{\theta }{\max}\int_{x}P_{r}(x)\log P_{G}(x;\theta )dx\\ & =\arg\underset{\theta }{\min}\int P_{r}(x)\log\frac{P_{r}(x)}{P_{G}(x;\theta )}dx \end{array}$$

The last equation called Kullback–Leibler (*KL*) divergence, which is commonly used to measure the difference between two distributions, is calculated as

(6) $$KL(P_{r}||P_{G})=E_{x\sim P_{r}}[\log\frac{P_{r}(x)}{P_{G}(x)}]=\int P_{r}(x)\log\frac{P_{r}(x)}{P_{G}(x)}dx$$

However, *KL* divergence is asymmetric. Therefore, one may change *KL* divergence into *JS* divergence, where GAN is introduced to judge how close the two distributions are. *JS* divergence can be expressed as

(7) $$JS(P_{r}||P_{G})=\frac{1}{2}KL(P_{r}||\frac{P_{r}+P_{G}}{2})+\frac{1}{2}KL(P_{G}||\frac{P_{r}+P_{G}}{2})$$

The value function *V*(*G, D*) is given by

(8) $$\begin{array}{cc} \underset{G}{\min}\;\underset{D}{\max}V(G,D)= & E_{x\sim P_{r}}[\log(D(x))]\\ & +E_{z\sim P_{z}}[\log(1-D(G(z)))] \end{array}$$

This is a maximum and minimum optimization problem and can be divided into two optimization problems:

Optimize *D*:

(9) $$\begin{array}{cc} \underset{D}{\min}V(D,G)= & -E_{x\sim P_{r}}[\log(D(x))]\\ & -E_{z\sim P_{z}}[\log(1-D(G(z)))] \end{array}$$

Optimize *G*:

(10) $$\underset{G}{\min}V(G,D)=E_{z\sim P_{z}}[\log(1-D(G(z)))]$$

The above is the original GAN, which can be seen as an approximation between two data spaces. Based on this spirit, an unsupervised adversarial discriminative domain adaptation framework is proposed. It assumes the source data *X_s_* and source label *Y_s_* is drawn from a source domain distribution *P_s_(x,y)*, while the target data *X_t_* is drawn from target domain distribution *P_t_(x,y)*, whose target label *Y_t_* is nonobservable. Based on these conditions, a source classifier can be built by seeking a source representation mapping *M_s_* along with a source classifier *C_s_*. The loss function of the source model can be defined as

(11) $$\begin{array}{cc} \underset{M_{s},C}{\min}L_{cls}(X_{s},Y_{s})= & E_{\left(x_{s},y_{s})\sim (X_{s},Y_{s}\right)}\\ & -\sum_{k=1}^{K}1_{\left[k=y_{s}\right]}\log C(M_{s}(X_{s})) \end{array}$$

where *1*_[*k=ys*]_ is an equation whose value equals to 1 when *k* = *y_s_* or 0 otherwise.

The goal is to learn a target representation *M_t_* and classifier *C_t_* that performs well in the target dataset. According to the domain adaptation theory, one should regularize the learning of the source and the target mappings, making the source model adapt to the usage of the target dataset. This minimizes the distance between source mapping distribution *M_s_*(*X_s_*) and target mapping distribution *M_t_*(*X_t_*) as much as possible. After this, the source classification model *C_s_* can be directly used to the target representations. To avoid learning a separate classifier, one can set *C_s_* = *C_t_*.

According to the original GAN theory, the source mapping distribution *M_s_*(*X_s_*) can be treated like real data, and the target mapping distribution *M_t_*(*X_t_*) can be seen like generated data. Therefore, an extra domain discriminator *D* is needed to distinguish whether the data comes from the source or the target domain. The loss of *D* can be written as

(12) $$\begin{array}{rr} \underset{D}{\min}\;L_{adv_{D}}(X_{s},X_{t},M_{s},M_{t})= & -E_{x_{s}\sim X_{s}}[\log D(M_{s}(X_{s}))]\\ & -E_{x_{t}\sim X_{t}}[\log(1-D(M_{t}(X_{t})))] \end{array}$$

The loss of target mapping can be defined as

(13) $$\underset{M_{s},M_{t}}{\min}\;L_{adv_{M}}(X_{s},X_{t},D)=E_{x_{t}\sim X_{t}}[\log D(M_{t}(X_{t}))]$$

## 3. Experiments

**Figure 2:**
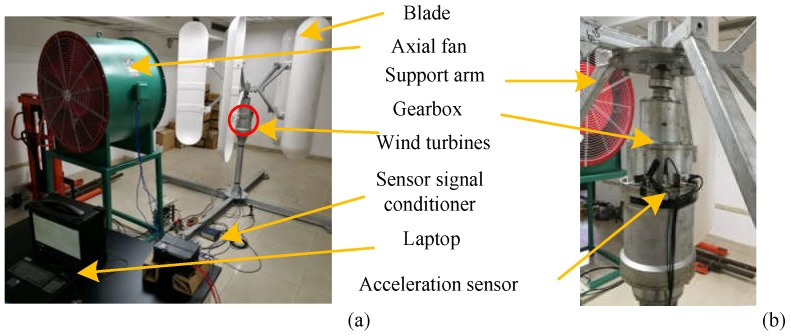
Wind turbine experimental setup: (**a**) components and architecture of the experimental setup; (**b**) position of the sensors.

To verify the effectiveness of the proposed GTL method algorithm, an experimental setup was installed as shown in Figure 2. In this experiment, as shown in Figure 2a, wind power was supplied by an axial flow fan (SWF-1-10), which drove the blades on the wind turbine (RCVA-3000) to rotate and then generate electricity. Three acceleration sensors and one acoustic emission sensor were mounted on the machine (RCVA-3000) near the gearbox. The acceleration sensor was connected to the sensor signal conditioner (PCB) and then connected to the high-speed data acquisition card (SQI, max sampling frequency: 1 MHz). Finally, the card was connected to the computer via a USB cable. A partial view of how the sensor was positioned on the wind turbine is shown in Figure 2b. During the experiment, we controlled the axial fan to move at different speeds. The sampling time for one acquisition was 20 s. The frequency converter that controlled the speed of the axial fan was set to 50 Hz. The axial fan drove the blades and generated the vibration signals that was acquired by the acceleration sensor. Each step of the faulty condition experiment was repeated 10 times to make sure there was enough data.

**Figure 3:**
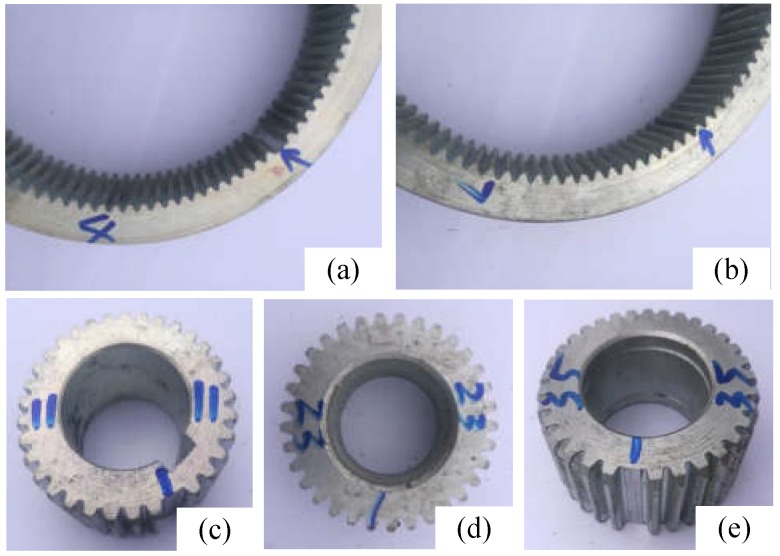
Five failure modes in the experiments: (**a**) ring gear with missing tooth; (**b**) ring gear with crack; (**c**) sun gear with missing tooth; (**d**) planetary gear root crack; (**e**) planetary gear with missing tooth.

The validation dataset was collected from this experimental setup, and the faulty components was mainly in a gearbox, which composed of a ring gear, a sun gear, and three planetary wheels. Vibration data were collected using accelerometers, which were attached to the gearbox housing. The dataset consisted of three domains that acquired at different working loads: high load (10.5 Ω), middle load (1 Ω), and low load (0.1 Ω). Each domain contained six categories (normal and five other faulty conditions), each of which had 1000 samples. The faulty data consisted of five conditions and two faulty types: missing tooth and crack (width 0.5 mm, depth 0.3 mm). The details are shown in Table 1. The data collected at different loads are called domain H, M, and L with the sampling frequency of 100 kHz. The corresponding faulty modes are shown in Figure 3.

## 4. Results and Discussion

In this section, the efficacy of the proposed GTL algorithm is evaluated using the wind turbine dataset, which was collected at different working loads. It is meant to make the model adapt to varying working loads but only train with samples from one working load.

In the following subsections, data preprocessing and network parameters are first given. Then, the performance of the GTL method is presented and compared to other algorithms. Network visualization is also introduced.

### 4.1. Data Preprocessing and Network Parameters

**Figure 4:**
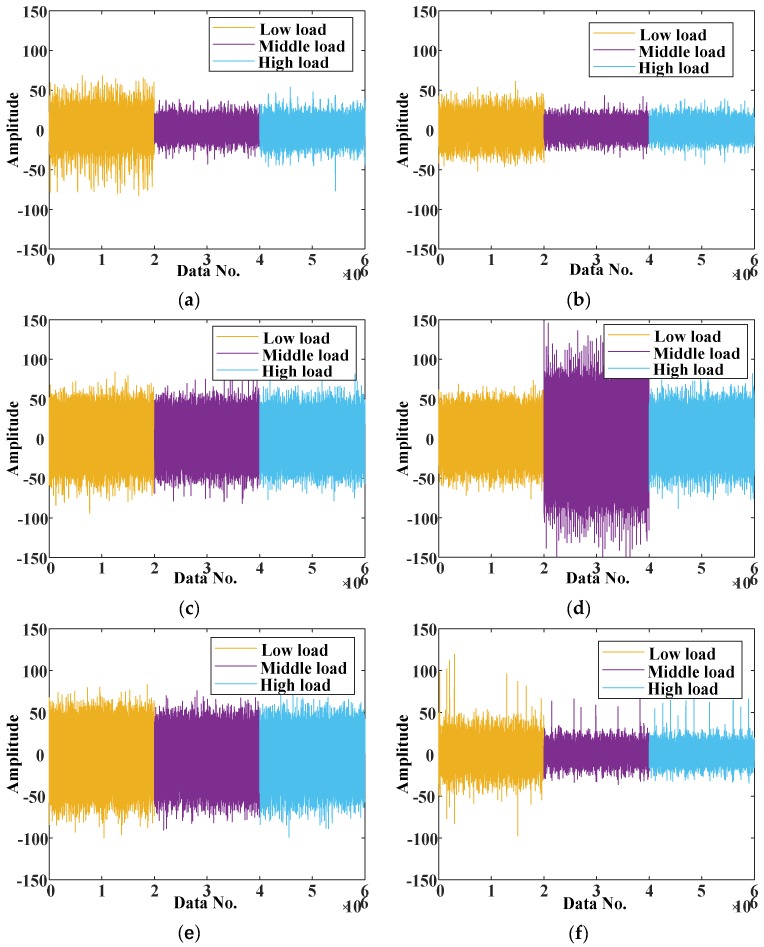
The raw signals: (**a**) C0 condition; (**b**) C1 condition; (**c**) C2 condition; (**d**) C3 condition; (**e**) C4 condition; (**f**) C5 condition.

**Figure 5:**
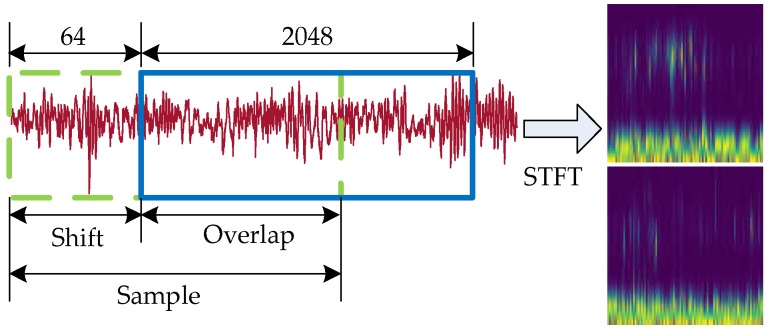
Data preprocess using data augmentation with overlap.

In this experiment, original signals under different load conditions, as shown in Figure 4, were collected from vibration accelerometers with 100k Hz sample frequency. We downsampled to 2500 Hz and chose every 2048 points as a sample. We also used a simple data augmentation trick, shown in Figure 5, to solve the problem of inadequate data, with the shift length of 64. After that, every sample was transformed by a short-time Fourier transformation with 128 window length from the time domain to the time–frequency domain so that it can feed into a two-dimensional convolutional neural network.

The architecture of the feature extractor used in this experiment consisted of two convolutional and pooling layers followed by two fully connected layers. The size of the convolutional kernel was 5 × 5, the pooling type was max pooling with kernel size of two, and the activation function was ReLU.

Moreover, the dropout and batch normalization operations were introduced in this net. A two-dimensional dropout operation was used after the first convolutional layer to add some noise to make the model more robust. Batch normalization, an operation that can help to accelerate the training process, was added right after the convolutional layers and after fully connected layers except before the last fully connected layer. The domain discriminator was composed of three fully connected layers. The number of neurons in each layer was 500, 500, and 2. The parameters of these nets are detailed in Table 2. The optimizer was an Adam stochastic optimization algorithm with hyperparameters *β_1_* = 0.5, *β_2_* = 0.9, except for the source feature extractor, which had set hyperparameters *β_1_* = 0.9, *β_2_* = 0.999. The learning rate of both the feature extractor and the domain discriminator was 0.0001. The experiments were implemented using the Pytorch toolbox of Facebook [30].

### 4.2. Accuracy across Different Load Domains

**Figure 6:**
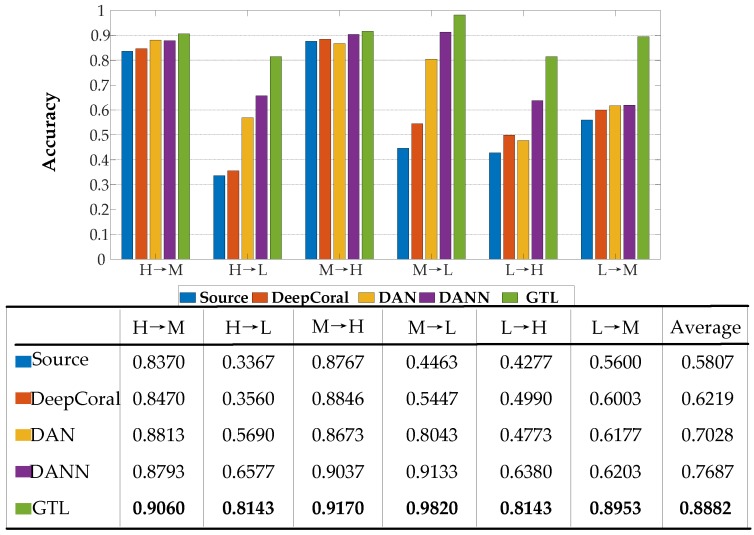
Accuracy (%) of the GTL method across three working load domains compared with four other methods.

As shown in Figure 6, classification of target domain data directly using the source net was not effective in whole transfer tasks except for the transfer tasks between the high working load domain (H) and the middle working load domain (M). This proves that models trained in one working condition are not suitable for classifying the data draw from different working conditions. Nevertheless, the reason the tasks between H and M could reach up to 83% might be because the distributions of the data draw from these two domains were far closer than the others. Besides, DeepCoral [31], DAN [32], and DANN performed poorly in these domain transfer tasks, with average accuracy around 62.19%, 70.28%, and 76.87%, respectively. Compared with the other three transfer learning methods, the GTL method performed significantly better in every task. The accuracy of the GTL method in the six domain transfer tasks were 0.9060, 0.8143, 0.9170, 0.9820, 0.8143, and 0.8953, respectively. These results suggest that the proposed domain adaptation method can significantly improve the fault diagnosis performance for wind turbine gearbox under different working conditions.

The transfer tasks between H and M, all methods showed up to 80% accuracy, and the GTL method was only around 7% and 4% higher than the other methods in task H→M and task M→H, respectively. However, when adapting H→L and L→H, the accuracy of the proposed GTL method was at least 15.66% and 17.63% better than the other methods. The average accuracy of 0.8882 in the whole transfer task with the GTL algorithm was far better than the other methods.

As a limited tool, transfer learning cannot transfer knowledge between any two domains, and its performance is significantly affected by the similarity of the source and the target domain data. The more obvious the amplitude difference, the more difficult is the classification. Combining the raw signals shown in Figure 4, the amplitude of each load under various conditions seems erratic and random. However, in our experiments, inferred from the above results (shown in Figure 6), the tasks H→M (0.9060), M→H (0.9170), M→L (0.9820), and L→M (0.8953) indicate that the data of two adjacent domains were more similar than that of two separated domains. These results show that the amplitude information of each faulty condition is hidden in a potential subspace and cannot be directly observed, but the trained model can distinguish these regular patterns and extract them as features. Moreover, the tasks H→L (0.8143) and L→H (0.8143) also show the superiority of GTL in reducing distribution differences when the source and the target domains are distant.

**Figure 7:**
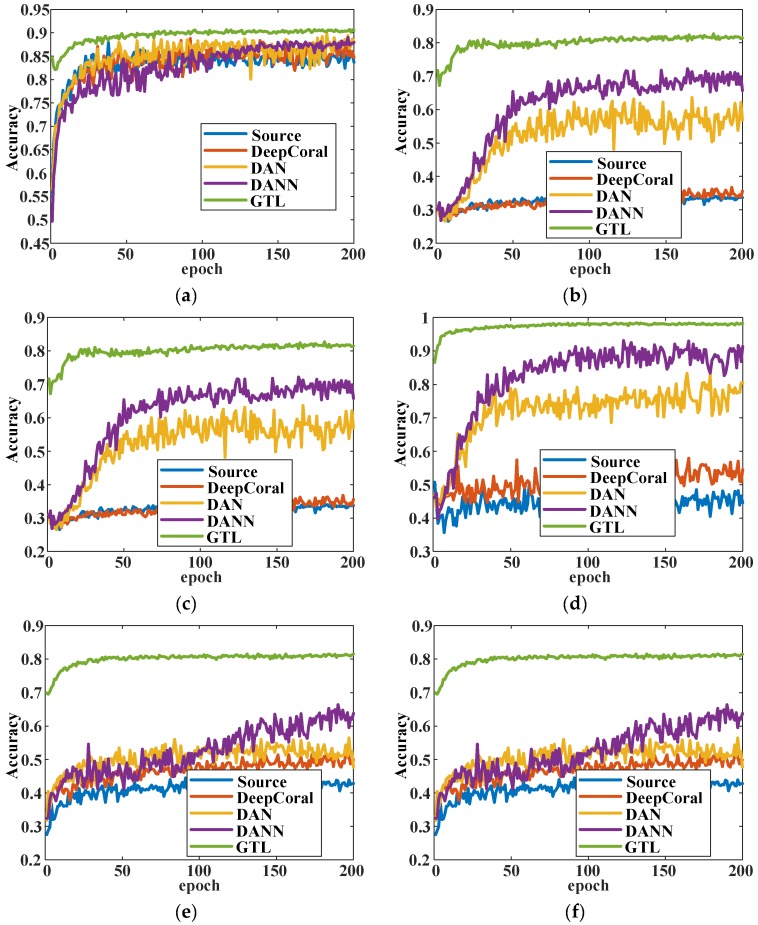
The changes of accuracy with epoch at six transfer tasks: (**a**) H→M; (**b**) H→L; (**c**) M→H; (**d**) M→L; (**e**) L→H; (**f**) L→M.

**Figure 8:**
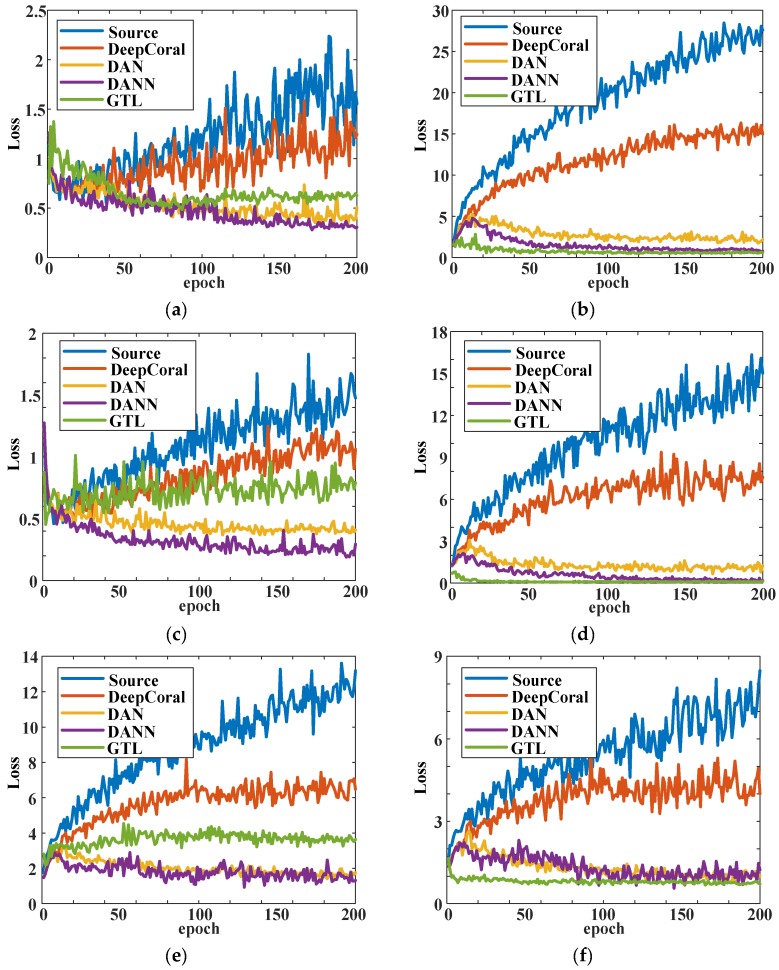
Test loss curve for six transfer tasks: (**a**) H→M; (**b**) H→L; (**c**) M→H; (**d**) M→L; (**e**) L→H; (**f**) L→M.

As one can see from Figure 7, in tasks H→M and M→H, the GTL method was slightly better than the other methods at the whole training process. For tasks where data distributions were distant, i.e., H→L, L→H, M→L, and L→M, the GTL showed a powerful ability of domain adaptation that converged faster. It also had better classification performance that was higher than the other methods by at least 10%. Source and DeepCoral showed poor performance in these four tasks and became stable at around the 50th epoch. DAN and DANN performed better than these two methods but still converged slower than the GTL method. The proposed GTL method not only performed better than the other methods but also converged faster at some transfer tasks. As the accuracy trend shows, it became stable at around the 20th epoch. The loss trend for the six transfer tasks are shown in Figure 8. As there was no training indicator to guide the adversarial training, the test loss curve was chosen to represent the convergence trend. However, the precision and recall significantly influenced the test loss. As can be seen in Figure 8a,c,e, the test loss of GTL was higher than DAN and DANN, as was the accuracy. For instance, at the transfer task L→H (Figure 8e), the accuracy of GTL (0.8143) was higher than the others, but the precision (0.5044) and recall (0.3340) were far lower than others. As a result, the test loss of GTL was higher than DAN and DANN. However, at distant domain transfer tasks, the GTL converged faster than the other four methods. Another interesting finding was that the accuracy of GTL in the first epoch was better than all the other algorithms, with all six tasks achieving over 70%. This proves that adversarial domain adaptation training can easily find better initial parameters for optimization. It should be noted that the methods that were compared have the same net architecture and hyperparameters. The loss function of DAN and DeepCoral is negative log likelihood loss, while the loss function is cross-entropy loss for the other methods.

### 4.3. Precision and Recall Performance Evaluation

In the machine learning field, precision and recall rate are two of the most commonly used indicators for model performance. To make further evaluation, the precision and recall rate were introduced to analyze the proposed method. The calculation of precision and recall rate of each *n*-th category can be presented as

(14) $$precision(n)=\frac{TP}{TP+FP}*100\%$$

(15) $$recall(n)=\frac{TP}{TP+FN}*100\%$$

where *TP* means the number of correctly identified labels, *FP* means the number of incorrectly identified labels, *FN* means the number of incorrectly identified labels that do not belong to the category *n*.

The precision and recall rates of each category in all cross-domain tasks are detailed in Table 3 and Table 4. As one can see from Table 3, the precision of ring gear with missing tooth and sun gear with missing tooth were higher than 90% in all domain transfer tasks, which means that each sample belonging to these two categories was almost accurately identified. For the ring gear crack condition, the proposed method had low precision in tasks H→L, L→H, and L→M, with 59.84%, 54.49%, and 70.18%, respectively. For the planetary wheel full broken tooth condition, the precision in tasks H→M and H→L were 77.75% and 79.01%, respectively. For the normal condition, precision in tasks H→L, M→H, and L→H were 61.60%, 79.07%, and 50.44%, respectively. These results mean that about 30%–50% fault alarms of these fault conditions were unreliable. Furthermore, the precision rates of the other conditions were in the range of 80%–90%.

As can be seen in Table 4, the recall of ring gear full broken tooth and sun gear full broken tooth reached above 90% in whole adaptation tasks, which means that there were about 10% missing alarms. The category of ring gear crack had an inferior recall in the transfer task H→M, H→L, M→H, and L→H, with 78.80%, 73.00%, 74.60%, and 77.40%, respectively. The recall of planetary wheel crack in task H→L was 78.20%. However, for the normal condition, the GTL method had very low recall when the transfer was from high load domain to low load domain and from low load domain to high load domain, with 45.60% and 33.40%, respectively. This means that up to half the number of this faulty condition was not detected.

### 4.4. Network Visualization

**Figure 9:**
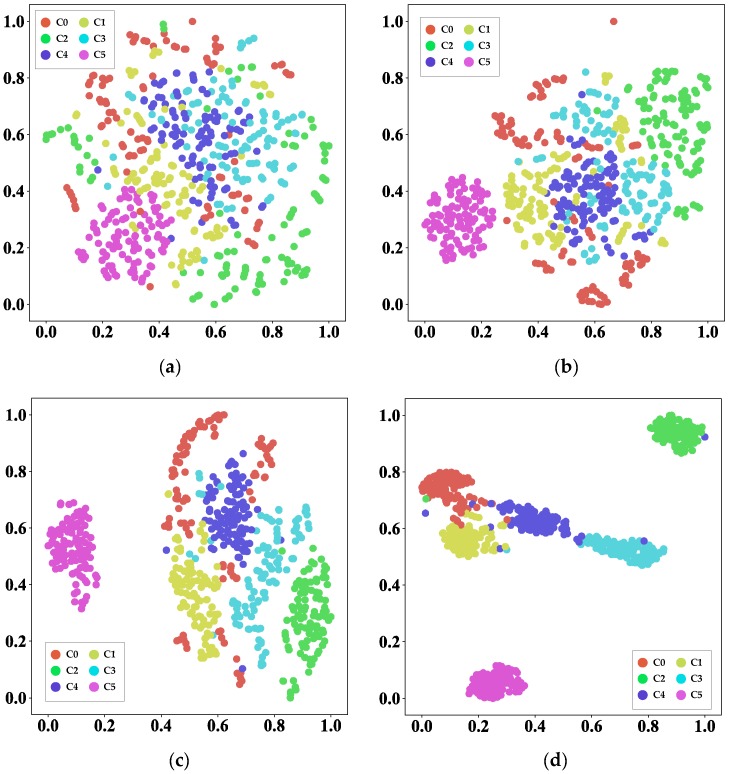

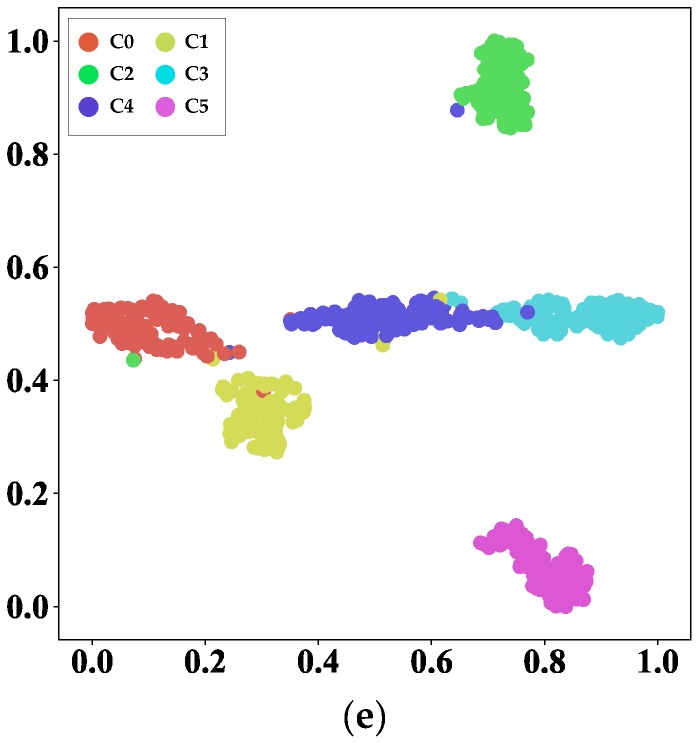
Feature visualization via t-distributed stochastic neighbor embedding (t-SNE) for raw data and all layers in task M→L: (**a**) raw data; (**b**) convolutional layer 1; (**c**) convolutional layer 2; (**d**) fully connected layer 1; (**e**) fully connected layer 2.

All deep models are seen like a black box, and nobody can tell how it operates. This work tried to take advantage of data visualization to explain why the proposed GTL method could achieve such a significant performance for wind turbine gearbox under different working loads. The visualization method used was t-distributed stochastic neighbor embedding (t-SNE) [33]. This is a dimension reduction method that can reduce the dimension of data and also retain the difference between samples. The transfer task M→L was taken as an example, and the visualizations of raw data and all layers are detailed in Figure 9.

There are some noteworthy findings. First, the six categories of raw data were evenly distributed and indivisible before adaptation. After training, the distribution of data began to disperse after convolutional layer 1. Then, each sample belonging to *n* started to cluster with each other. After a full training process, every category could be easily distinguished. Figure 9e shows that faulty conditions 0, 3, and 4 still had some overlapped region, which proved that this model misclassified these three conditions and caused some accuracy loss.

## 5. Conclusions

In this paper, a generative transfer learning method based on generative adversarial network is reported for domain adaptation in fault diagnosis of a wind turbine gearbox under various working loads. The GTL method draws lessons from GAN and can be divided into three steps: training a source network; building a target network with the same architecture as that of the source and introducing a discriminator to distinguish the domain of the labels; and training the target net using the reverse label. The performance of the proposed GTL was studied and experimentally validated. The main attributes of the GTL method are as follows. (1) It has good performance. As an unsupervised domain adaptation method, the GTL method beat the peer methods and showed significant performance in the wind turbine dataset. (2) It has faster convergence speed at distant domain transfer tasks. Compared with other peer methods, GTL converged faster at distant domain transfer tasks. Although the GTL method presents excellent performance, drawbacks still exist as follows. (1) It has low accuracy during distant transfer tasks. The accuracy of transfer tasks between high and low working load domains was below 90%. (2) It has low precision and recall rate during distant transfer tasks. In the experiments, the precision and recall in some conditions were even less than 60%. Therefore, the way to further enhance the adaptability of the GTL method in data between domains where distributions are distant will be our future work direction.

## Figures and Tables

**Table 1:** Details of the faulty patterns in the experiments.

Pattern No.	Fault Location	Degree of Failure
**C0**	Ring gear	Full broken tooth
**C1**	Ring gear	Crack (width 0.5 mm, depth 0.3 mm)
**C2**	Sun gear	Full broken tooth
**C3**	Planetary wheel	Crack (width 0.5 mm, depth 0.3 mm)
**C4**	Planetary wheel	Full broken tooth
**C5**	Normal	Normal

**Table 2:** Details of the network and parameters.

	Layer type	Kernel	Stride	Channel	Activation Function
**Feature extractor**	Conv1	5 × 5	1	20	ReLU
	pooling 1	2 × 2	None	20	
	Conv2	5 × 5	1	50	ReLU
	pooling 2	2 × 2	None	50	
	fc 1	500		1	ReLU
**Classifier**	fc 2	6		1	Softmax
**Domain discriminator**	fc 1	500		1	ReLU
	fc 2	500		1	ReLU
	fc 3	2		1	Log Softmax

**Table 3:** Precision of the proposed GTL method.

Category Labels^a^	C0(%)	C1(%)	C2(%)	C3(%)	C4(%)	C5(%)
H→M	98.06	80.33	98.23	98.20	77.75	94.86
H→L	97.73	59.84	99.02	98.24	79.01	61.60
M→H	96.22	85.99	98.63	99.60	93.23	79.07
M→L	98.99	97.76	100.00	99.80	93.25	100.00
L→H	98.05	54.49	89.88	94.77	91.53	50.44
L→M	95.73	70.18	97.86	97.48	89.14	97.67

**Table 4:** Recall of the proposed GTL method.

Category Labels^a^	C0(%)	C1(%)	C2(%)	C3(%)	C4(%)	C5(%)
H→M	91.20	78.80	99.00	97.20	90.40	87.00
H→L	94.60	73.00	99.80	78.20	97.40	45.60
M→H	96.60	74.60	99.60	95.80	91.20	92.40
M→L	97.00	99.80	100.00	94.60	98.80	99.00
L→H	98.80	77.40	98.60	97.20	83.20	33.40
L→M	93.60	89.40	100.00	99.80	87.40	67.00

^a^ Category labels represent the six conditions: C0 = ring gear full broken tooth, C1 = ring gear crack, C2 = sun gear full broken tooth, C3 = planetary wheel full broken tooth, C4 = planetary wheel full broken tooth, C5 = normal condition.

## References

[1] Zhang X.Y., Liang Y.T., Zhou J.Z., Zang Y. (2015). A novel bearing fault diagnosis model integrated permutation entropy, ensemble empirical mode decomposition and optimized SVM. Measurement.

[2] Wang T.Z., Qi J., Xu H., Wang Y.D., Liu L., Gao D.J. (2016). Fault diagnosis method based on FFT-RPCA-SVM for cascaded-multilevel inverter. ISA Trans..

[3] Li W., Zhu Z.C., Jiang F., Zhou G.B., Chen G.A. (2015). Fault diagnosis of rotating machinery with a novel statistical feature extraction and evaluation method. Mech. Syst. Signal Process..

[4] Hao Y., Song L., Ren B., Wang H., Cui L. (2019). Step-by-step compound faults diagnosis method for equipment based on majorization-minimization and constraint SCA. IEEE ASME Trans. Mechatron..

[5] Cabrera D., Sancho F., Li C., Cerrada M., Sanchez R.V., Pacheco F., de Oliveira J.V. (2017). Automatic feature extraction of time-series applied to fault severity assessment of helical gearbox in stationary and non-stationary speed operation. Appl. Soft Comput..

[6] Lee K.P., Wu B.H., Peng S.L. (2019). Deep-learning-based fault detection and diagnosis of air-handling units. Build. Environ..

[7] Zhang S.H., Sun Z.Z., Wang M., Long J.Y., Bai Y., Li C. (2019). Deep fuzzy echo state networks for machinery fault diagnosis. IEEE Trans. Fuzzy Syst..

[8] Zhang S.H., Sun Z.Z., Li C., Cabrera D., Long J.Y., Bai Y. (2019). Deep hybrid state network with feature reinforcement for intelligent fault diagnosis of delta 3D printers. Ieee Trans. Ind. Inf..

[9] Cabrera D., Sancho F., Cerrada M., Sanchez R.V., Tobar F. (2018). Echo state network and variational autoencoder for efficient one-class learning on dynamical systems. J. Intell. Fuzzy Syst..

[10] Shao H.D., Jiang H.K., Liu X.Q., Wu S.P. (2018). Intelligent fault diagnosis of rolling bearing using deep wavelet auto-encoder with extreme learning machine. Knowl. Based Syst..

[11] Sun C., Ma M., Zhao Z.B., Chen X.F. (2018). Sparse deep stacking network for fault diagnosis of motor. IEEE Trans. Ind. Inf..

[12] Shao H.D., Jiang H.K., Lin Y., Li X.Q. (2018). A novel method for intelligent fault diagnosis of rolling bearings using ensemble deep auto-encoders. Mech. Syst. Signal Process..

[13] Long J.Y., Sun Z.Z., Li C., Hong Y., Bai Y., Zhang S.H. (2019). A novel sparse echo autoencoder network for data-driven fault diagnosis of delta 3-D Printers. IEEE Trans. Instrum. Meas..

[14] Long J.Y., Sun Z.Z., Panos M.P., Hong Y., Zhang S.H., Li C. (2019). A hybrid multi-objective genetic local search algorithm for the prize-collecting vehicle routing problem. Inf. Sci..

[15] Li C., Oliveira J.L.V.D., Lozada M.C., Cabrera D., Sanchez V., Zurita G. (2019). A systematic review of fuzzy formalisms for bearing fault diagnosis. IEEE Trans. Fuzzy Syst..

[16] Long J.Y., Zhang S.H., Li C. (2019). Evolving deep echo state networks for intelligent fault diagnosis. IEEE Trans. Ind. Inf..

[17] Pan S.J., Yang Q. (2010). A survey on transfer learning. IEEE Trans. Knowl. Data Eng..

[18] Li C., Cerrada M., Cabrera D., Sanchez R.V., Pacheco F., Ulutagay G., de Oliveira J.V. (2018). A comparison of fuzzy clustering algorithms for bearing fault diagnosis. J. Intell. Fuzzy Syst..

[19] Guo L., Lei Y.G., Xing S.B., Yan T., Li N.P. (2019). Deep convolutional transfer learning network: A new method for intelligent fault diagnosis of machines with unlabeled data. IEEE Trans. Ind. Electron..

[20] Lu W.N., Liang B., Cheng Y., Meng D.S., Yang J., Zhang T. (2017). Deep model based domain adaptation for fault diagnosis. IEEE Trans. Ind. Electron..

[21] Han T., Liu C., Yang W.G., Jiang D.X. (2019). Deep transfer network with joint distribution adaptation: A new intelligent fault diagnosis framework for industry application. ISA Trans..

[22] Guo J.W., Wu J.P., Sun Z.Z., Long J.Y., Zhang S.H. (2019). Fault diagnosis of delta 3D printers using transfer support vector machine with attitude signals. IEEE Access..

[23] Shao S.Y., McAleer S., Yan R.Q., Baldi P. (2018). Highly-accurate machine fault diagnosis using deep transfer learning. IEEE Trans. Ind. Inf..

[24] Wen L., Gao L., Li X.Y. (2019). A new deep transfer learning based on sparse auto-encoder for fault diagnosis. IEEE Trans. Syst. Mancybern. Syst..

[25] Tong Z., Li W., Zhang B., Jiang F., Zhou G.B. (2018). Bearing fault diagnosis under variable working conditions based on domain adaptation using feature transfer learning. IEEE Access..

[26] Goodfellow I., Pouget-Abadie J., Mirza M., Xu B., Warde-Farley D., Ozair S., Courville A., Bengio Y. (2014). Generative adversarial nets. Adv. Neural Inf. Process. Syst..

[27] Ganin Y., Ustinova E., Ajakan H., Germain P., Larochelle H., Laviolette F., Mario M., Lempitsky V. (2016). Domain-adversarial training of neural networks. J. Mach. Learn. Res..

[28] Cabrera D., Sancho F., Long J.Y., Sánchez R.V., Zhang S.H., Cerrada M., Li C. (2019). Generative adversarial networks selection approach for extremely imbalanced fault diagnosis of reciprocating machinery. IEEE Access..

[29] Ioffe S., Szegedy C. (2015). Batch normalization: Accelerating deep network training by reducing internal covariate shift. arXiv.

[30] Paszke A., Gross S., Chintala S., Chanan G., Yang E., DeVito Z., Ling Z.M., Desmaison A., Antiga L., Lerer A. Automatic differentiation in PyTorch. Proceedings of the 31st Conference on Neural Information Processing Systems (NIPS 2017).

[31] Sun B.C., Saenko K. Deep coral: Correlation alignment for deep domain adaptation. Proceedings of the European Conference on Computer Vision.

[32] Long M.S., Cao Y., Wang J.M., Jordan M.I. Learning transferable features with deep adaptation networks. Proceedings of the International Conference on Machine Learning.

[33] Maaten L.V.D., Hinton G. (2008). Visualizing data using t-SNE. J. Mach. Learn. Res..

